# The effect of Guipiheji (Guipi Mixture) combined with ferrous succinate tablets on improving iron deficiency anemia

**DOI:** 10.3389/fmed.2026.1723257

**Published:** 2026-01-29

**Authors:** Mingxu Chen, Xinzheng Wei, Hao Lu, Shengwei Hou, Jianqiao Yang, Yi Zhang, Yifang Wang, Ziqing Yang

**Affiliations:** 1 Department of Rehabilitation Medicine, Gongshu District People’s Hospital of Integrated Traditional Chinese and Western Medicine, Hangzhou, Zhejiang, China; 2 Department of Cardiology, Gongshu District People’s Hospital of Integrated Traditional Chinese and Western Medicine, Hangzhou, Zhejiang, China; 3 Department of Rehabilitation Medicine, Gongshu District People’s Hospital of Integrated Traditional Chinese and Western Medicine, Hangzhou, Zhejiang, China; 4 Department of Rehabilitation Medicine, Hangzhou Tianrui Hospital Co., Ltd, Hangzhou, Zhejiang, China

**Keywords:** ferrous succinate tablets, Guipiheji, iron deficiency anemia, symptom, traditional Chinese patent medicines

## Abstract

**Objective:**

The aim of this study is to investigate the effect of Guipiheji (Guipi Mixture) combined with ferrous succinate tablets on improving iron deficiency anemia (IDA).

**Methods:**

A retrospective analysis was conducted on 80 IDA patients admitted to Gongshu District People’s Hospital of Integrated Traditional Chinese and Western Medicine from November 2024 to May 2025. Participants were assigned to an observation group (*n* = 40, Guipi Mixture plus ferrous succinate) or a control group (*n* = 40, ferrous succinate alone). Pre- and post-treatment levels of hemoglobin (Hb), hematocrit (HCT), red blood cell count (RBC), mean corpuscular volume (MCV), serum iron (SI), total iron binding capacity (TIBC), and iron saturation were compared between groups. Symptom relief and adverse reactions post-treatment were also evaluated.

**Results:**

After treatment, levels of Hb, HCT, RBC, MCV, SI, iron saturation, and CD3^+^, CD4^+^, and CD8^+^ T-cells were significantly elevated in both groups post-treatment, with the observation group showing higher values than the control group (*P* < 0.05); TIBC levels decreased in both groups post-treatment, with the observation group exhibiting significantly lower values than the control group (*P* < 0.05). The effective rate of the observation group was higher than that of the control group (*P* < 0.05). Univariate logistic regression analysis showed that the severity of anemia (OR = 3.595, 95%CI: 1.139–11.350, *P* = 0.029) and pre-treatment hemoglobin level (OR = 0.933, 95%CI: 0.874–0.996, *P* = 0.037) were significantly associated with treatment response. After adjusting for variables with *P* < 0.10 in the univariate analysis (anemia severity, pre-treatment Hb, pre-treatment MCV) by incorporating them into a multivariate logistic regression model, none of the variables showed independent predictive value (*P* > 0.05).

**Conclusion:**

These results provide preliminary evidence that adding Guipiheji (Guipi Mixture) to ferrous succinate tablets is more effective in treating IDA. However, our study has some limitations. Specifically, we did not control for important confounding factors (including the severity of anemia, comorbidities, inflammation, concurrent medication use, menstrual blood loss, gastrointestinal malabsorption, renal function, and socioeconomic variables). Additionally, dietary intake, which may affect iron absorption and hematological outcomes, was not regulated in the research.

## Introduction

Iron deficiency anemia (IDA) refers to anemia caused by a decrease in the synthesis of hemoglobin in the human body due to iron deficiency, which in turn affects the production of red blood cells (RBC) (1, 2). IDA is characterized by low levels of RBC and hemoglobin concentration in the blood, low levels of transferrin in the serum, and relatively low levels of iron (2). IDA is the most common type of anemia worldwide and the only nutritional anemia that affects large populations in both developed and developing countries (1–3). Research has shown that iron deficiency exerts adverse effects on the regulation of specific immune system functions, such as immune cells and immune active factors, which in turn affect and regulate the body’s inflammatory levels (3, 4). Changes in iron levels can lead to changes in oxygen free radicals, causing oxidative damage (4).

Oral iron supplements are the cornerstone of treating IDA, but their adverse reactions can reduce compliance and lead to suboptimal efficacy (5). Guipiheji (Guipi Mixture) is processed from Guipi decoction, and its dosage form features a convenient dosage form and acceptable taste (6). Numerous clinical studies have been conducted on the application of Guipiheji (Guipi Mixture) in various diseases, all demonstrating that Guipiheji (Guipi Mixture) has advantages in improving symptoms and related indicators (7, 8). Si et al. (7) found through clinical research that the combination of Guipiheji (Guipi Mixture) and propafenone hydrochloride tablets has a better therapeutic effect on heart spleen deficiency type arrhythmia, which can further alleviate patients’ clinical symptoms and improve their heart function. Zhu et al. (8) found that the modified Guipiheji (Guipi Mixture) can effectively increase platelet count and Treg cells in patients with chronic immune thrombocytopenia and qi deficiency syndrome, and improve related immune dysfunction.

Guipiheji (Guipi Mixture) exerts significant efficacy in alleviating clinical symptoms of various diseases such as qi-blood deficiency, heart-spleen deficiency, and is widely used in hematological diseases (6–8). Due to adverse reactions in the treatment of IDA with Western medicine, patients have poor long-term acceptance. On the contrary, traditional Chinese medicine (TCM) can improve certain side effects while treating diseases, making it easier for patients to accept long-term treatment. Therefore, we hypothesize that for IDA patients with syndromes such as qi-blood deficiency, heart-spleen deficiency, the use of Guipiheji (Guipi Mixture) can be added to conventional Western medicine treatment in order to achieve better therapeutic effects than simple Western medicine treatment. Furthermore, in line with the contemporary understanding of IDA as a disorder involving systemic metabolic and immune disturbances of IDA as a disorder with systemic metabolic and immune implications, this study also aimed to evaluate the impact of the combined therapy on cellular immune function, by assessing T-lymphocyte subsets (CD3^+^, CD4^+^, CD8^+^), in addition to conventional hematologic and iron metabolism parameters.

## Materials and methods

Study design and patient identification: This retrospective cohort study was conducted at Gongshu District People’s Hospital of Integrated Traditional Chinese and Western Medicine. Patients were identified through the hospital’s electronic medical record (EMR) system using the International Classification of Diseases, Tenth Revision (ICD-10) (1) codes for iron deficiency anemia (D50.0, D50.8, and D50.9). Initial screening included all adult patients (aged 18–80 years) diagnosed with IDA between November 2024 and May 2025. All identified records were independently reviewed by two trained researchers (Xinzheng Wei and Hao Lu) to verify diagnostic consistency and eligibility based on the inclusion and exclusion criteria. Discrepancies were resolved through discussion with a third senior investigator (Shengwei Hou).

Western medicine diagnosis refers to the diagnostic criteria for IDA in the “Diagnosis and Efficacy Standards for Hematological Diseases” (1). (1) Anemia is characterized by small cell hypopigmentation, with hemoglobin (Hb) levels below 120 g/L in males, < 110 g/L in females, and < 100 g/L in pregnant women; Meanwhile, the mean corpuscular volume (MCV) is less than 80 fL, the mean corpuscular hemoglobin content is less than 27 pg, and the mean corpuscular hemoglobin concentration is less than 320 g/L; (2) There are obvious causes and clinical manifestations of iron deficiency; (3) Serum iron (SI) < 8.95 μmol/L, total iron binding capacity (TIBC) > 64.44 μmol/L; (4) Transferrin saturation < 15%; (5) Bone marrow iron staining showed that the iron in the bone marrow granules disappeared, and the iron content in the RBC was less than 15%; (6) RBC free erythrocyte protoporphyrin IX (FEP) > 0.9 μmol/L (whole blood), or blood zinc protoporphyrin (ZPP) > 0.96 μmol/L (whole blood), or FEP/Hb > 4.5 μg/g; (7) Serum ferritin < 12 μg/L; (8) Serum soluble transferrin receptor > 26.5 nmol/L; (9) Iron therapy is effective. To be diagnosed with IDA, one must meet the requirements of item 1 and any two or more of items (2) to (9).

TCM syndrome differentiation: In addition to the Western medical diagnosis, all patients enrolled in this study underwent TCM syndrome assessment by two certified TCM physicians with more than 10 years of clinical experience. The assessment was based on the classical TCM syndrome patterns associated with anemia, primarily qi-blood deficiency and heart-spleen deficiency. The diagnostic criteria were derived from the Chinese Internal Medicine textbook and clinical consensus, focusing on the following key symptoms and signs: (1) Qi-blood deficiency: Fatigue, pale or sallow complexion, palpitations, shortness of breath, dizziness, pale tongue, thready and weak pulse. (2) Heart-spleen deficiency: Palpitations, insomnia, forgetfulness, poor appetite, abdominal distension, loose stools, pale tongue with thin coating, thready and weak pulse. Patients were included in the observation group only if they met the diagnostic criteria for at least one of the above syndromes and were deemed suitable for treatment with Guipiheji (Guipi Mixture), which is classically indicated for syndromes of heart-spleen deficiency and qi-blood deficiency. Any disagreement between the two TCM physicians was resolved through discussion with a third senior TCM expert.

Application of diagnostic criteria in the study cohort: The diagnostic criteria listed above represent the comprehensive standard for IDA. In clinical practice at our center, the diagnosis is typically established using a combination of essential and readily available parameters. For all patients included in this study, the diagnosis was confirmed by satisfying criterion 1 (microcytic hypochromic anemia) plus at least two of the following routinely assessed items: clinical history of iron deficiency (criterion 2), serum iron < 8.95 μmol/L and total iron-binding capacity > 64.44 μmol/L (criterion 3), transferrin saturation < 15% (criterion 4), and serum ferritin < 12 μg/L (criterion 7). Specialized tests such as bone marrow iron staining (criterion 5), erythrocyte protoporphyrin (criterion 6), or serum soluble transferrin receptor (criterion 8) were performed only in cases of diagnostic uncertainty and were not routinely documented for all patients. Therefore, while the full diagnostic spectrum is presented, the eligibility of patients for this study relied primarily on the core hematologic and iron metabolism parameters consistently available in the electronic medical records.

Sample size estimation: As a retrospective cohort study, the sample size was determined by the number of eligible IDA patients who received either of the two treatment regimens during the study period (November 2024 to May 2025). No *a priori* sample size calculation was performed given the exploratory nature of this retrospective analysis. However, to evaluate the statistical reliability of the observed between-group difference in the primary outcome (post-treatment hemoglobin level), a *post hoc* power analysis was conducted using G*Power 3.1, based on the between-group difference in post-treatment hemoglobin reported in Table 1. With α = 0.05 and *n* = 40 per group, the achieved *post hoc* power was 0.87, indicating adequate statistical sensitivity for detecting a clinically meaningful between-group difference in the main hematologic outcome.

**TABLE 1:** Comparison of ORR between two groups.

Group	CR	PR	NR	ORR
Observation group (*n* = 40)	23 (57.5)	14 (35.0)	3 (7.5)	37 (92.5)
Control group (*n* = 40)	13 (32.5)	17 (42.5)	10 (25.0)	30 (75.0)
*P*				0.034

CR, complete remission; PR, partial relief; NR, no response; ORR, objective response rate.

### Inclusion criteria

- Meets the diagnostic criteria of Western medicine mentioned above;
- Age range: 18–80 years old;
- Complete clinical data.

### Exclusion criteria

- Patients with severe liver and kidney dysfunction;
- Patients with severe primary diseases of the heart, brain, lungs, endocrine system, and gastrointestinal tract;
- Severe anemia requiring blood transfusion treatment;
- Individuals with reduced whole blood cells.

Ferrous succinate tablets (manufacturer: Chengdu Aobang Pharmaceutical Co., Ltd.), oral, 0.1 g/tablet, 2–4 tablets per day. Treatment for 8 weeks.

Guipiheji (Guipi Mixture, Manufacturer: Zhengda Qingchunbao Pharmaceutical Co., Ltd.; 10 mL × 10 vials/box), taken orally, 10–20 mL once, 3 times a day. Treatment for 8 weeks.

Pharmacological Intervention: Patients in the control group orally received ferrous succinate tablets (manufacturer: Chengdu Aobang Pharmaceutical Co., Ltd.; specification: 0.1 g/tablet), 2–4 tablets each time, once daily, for a treatment course of 8 weeks. Patients in the observation group received the same dosage of ferrous succinate tablets as the control group, with the addition of Guipiheji (Guipi Mixture; Manufacturer: Zhengda Qingchunbao Pharmaceutical Co., Ltd.; approval number: National Medicine Permit No. Z33021078; specification: 10 mL × 10 vials/box). Guipiheji (Guipi Mixture) was administered orally, 10–20 mL each time, three times daily, for a treatment course of 8 weeks. Guipiheji (Guipi Mixture) is an oral liquid preparation based on the classic formula “Guipi Decoction.” Its specific composition (according to the drug instructions and production standards) is detailed in Table 2.

**TABLE 2:** Composition of Guipiheji (Guipi Mixture) used in this study.

Chinese name	Latin name (Botanical name)	Family	Part used	Amount per dose (g) (or per 100 mL)
Huangqi	Astragalus mongholicus Bunge	Fabaceae	Root	15
Baizhu	Atractylodes macrocephala Koidz.	Asteraceae	Rhizome	15
Danggui	Angelica sinensis (Oliv.) Diels	Apiaceae	Root	9
Dangshen	Codonopsis pilosula (Franch.) Nannf.	Campanulaceae	Root	15
Fuling	Poria cocos F.A.Wolf	Polyporaceae	Sclerotium	15
Yuanzhi	Polygala tenuifolia Willd.	Polygalaceae	Root	9
Suanzaoren	*Ziziphus jujuba* subsp. *spinosa* (Bunge) J.Y.Peng, X.Y.Li & L.Li	Rhamnaceae	Seed	15
Longyanrou	Dimocarpus longan Lour.	Sapindaceae	Aril	15
Muxiang	Aucklandia lappa (Decne.) Decne.	Asteraceae	Root	9
Gancao	Glycyrrhiza uralensis Fisch. ex DC.	Fabaceae	Root and Rhizome	6
Dazao	Ziziphus jujuba Mill.	Rhamnaceae	Fruit	3
Shengjiang	Zingiber officinale Roscoe	Zingiberaceae	Rhizome	3

Data collection: (1) Collect baseline data of patients, including gender, age, body mass index (BMI), degree of anemia, and cause of anemia. Severity grading of anemia: Based on the concentration of Hb, it is divided into mild (91–120 g/L for males and 81–105 g/L for females), moderate (61–90 g/L for males and 61–80 g/L for females), severe (31–60 g/L for males and 41–60 g/L for females), and extremely severe (< 31 g/L for males and < 41 g/L for females). (2) RBC parameter indicators, including changes in Hb, hematocrit (HCT), RBC, MCV levels. (3) Iron metabolism indicators, including iron, TIBC, and iron saturation. (4) Clinical efficacy, complete remission (CR): anemia symptoms significantly improved, Hb increased > 30 g/L; Specifically, the Hb of adult males should be > 130 g/L, and the Hb of adult females should be > 120 g/L (pregnant women > 110 g/L). Partial relief (PR): Anemia symptoms have significantly improved, although Hb levels are lower than normal, Hb levels have increased by more than 15 g/L. No response (NR): There is no significant improvement in anemia symptoms, and Hb levels are not elevated or slightly elevated. The objective response rate (ORR) is calculated as follows: (number of CR cases + number of PR cases)/total number of cases × 100%. (5) Adverse reactions include nausea, upper abdominal discomfort, phlebitis, decreased appetite, facial flushing, constipation, etc.

Timing of post-treatment assessment: All post-treatment laboratory tests (hematologic, iron-metabolic, and immunologic parameters) were performed within 3 days after completing the 8-week treatment course.

Blood sampling conditions: Venous blood samples were collected after an overnight fast (≥ 8 h) to minimize dietary influence on serum iron and related markers.

Laboratory instruments and assay methods: (1) Complete blood count (Hb, HCT, RBC, MCV) was measured using a Sysmex XN–9000 automated hematology analyzer (Sysmex Corporation, Kobe, Japan). (2) Serum iron (SI), total iron-binding capacity (TIBC), and iron saturation were determined by colorimetric assay (Roche Cobas c702, Roche Diagnostics, Basel, Switzerland). (3) Serum ferritin was measured by chemiluminescent immunoassay (Abbott Architect i2000SR, Abbott Laboratories, Chicago, IL, United States). (4) T-cell subsets (CD3^+^, CD4^+^, CD8^+^) were analyzed by flow cytometry (BD FACSCanto II, BD Biosciences, San Jose, CA, United States) using commercially available monoclonal antibody panels.

Additional iron-status markers: Although not included as primary endpoints, serum ferritin and reticulocyte hemoglobin content (Ret-He) were also recorded when available. Ferritin was assessed as described above; Ret-He was obtained from the Sysmex XN-9000 reticulocyte channel.

Assessment of symptom relief: Symptom improvement was evaluated using a structured questionnaire that included the following IDA-related symptoms: fatigue, dizziness, palpitations, pale complexion, and shortness of breath. Each symptom was scored on a 4-point Likert scale (0 = absent, 1 = mild, 2 = moderate, 3 = severe). The total symptom score was calculated at baseline and after treatment. A reduction of ≥ 50% in the total score was defined as “clinically significant improvement.” This assessment was performed by the same clinician who was blinded to the treatment allocation.

Adverse reaction monitoring and documentation: Adverse reactions were systematically collected through retrospective review of the electronic medical records (EMRs). All documented adverse events related to the treatment during the 8-week intervention period were extracted from clinical notes, follow-up visit records, and patient self-reports recorded in the EMRs. Adverse reactions of interest included nausea, epigastric discomfort, phlebitis, loss of appetite, facial flushing, and constipation, as predefined in the study protocol. Events were included regardless of whether they were explicitly attributed to the study medications by the treating physician, as long as they occurred during the treatment period. This approach reflects real-world clinical documentation practices and ensures comprehensive capture of safety data within the limitations of a retrospective design.

Assessment of potential confounding variables and adjustment strategy: To evaluate the comparability between the two treatment groups beyond basic demographics, we extracted data on comorbidities (e.g., hypertension, diabetes mellitus, gastrointestinal disorders, and malignant tumors), concurrent medications (e.g., proton pump inhibitors, vitamin C, other hematinic agents used during the 8-week treatment period), and nutritional status (approximated by BMI and documented dietary advice at enrollment). These variables were compared between—groups (Table 2) to identify potential imbalances that might affect hematologic outcomes.

Assessment of treatment adherence and consistency: To evaluate adherence to the prescribed treatment regimens, we extracted pharmacy dispensing records and medication administration documentation from the electronic medical records for both ferrous succinate tablets and Guipiheji (Guipi Mixture) (observation group). Adherence was approximated by calculating the medication possession ratio (MPR) based on the total amount of medication dispensed relative to the prescribed dosage over the 8-week treatment period. An MPR of ≥ 80% was considered indicative of good adherence. For oral iron therapy, adherence was further supported by routine clinical notation of “taking medication as prescribed” in follow-up visit notes, which was documented for all included patients. The consistency of Guipiheji (Guipi Mixture) administration (dosage and frequency) was verified against the standard regimen described in the Methods. Any documented deviations (e.g., dose adjustment, early discontinuation) were recorded. Both groups were comparable in terms of adherence assessment, and no patient was excluded due to documented non-adherence in the medical records.

Data source verification and bias reduction:Data were extracted from the hospital’s structured EMR modules, including laboratory, pharmacy, and clinical notes. To ensure accuracy, key variables (e.g., hemoglobin, serum iron, medication records) were cross-verified with original laboratory reports and prescription logs. To minimize selection bias, we consecutively included all eligible patients during the study period without subjective exclusion. To reduce information bias, data extraction followed a predefined standardized form, and abstractors were blinded to the study hypothesis during the initial data collection phase. Regular consistency checks were performed on 20% of randomly selected records, yielding a data concordance rate of > 95%.

Handling of missing data: This retrospective study relied on data extracted from electronic medical records, which may contain incomplete entries. Prior to analysis, all variables of interest (e.g., laboratory parameters, demographic information) were screened for missingness. For baseline characteristics and outcome variables essential to the study (including hemoglobin, hematocrit, RBC indices, iron metabolism markers, and T-cell subsets), records with missing data in any of these key fields were excluded from the final analysis to ensure data consistency. No imputation methods were applied for missing values. The number of excluded records due to missing data is reported in the Results section. This approach aligns with a complete-case analysis strategy commonly used in retrospective cohort studies when missingness is minimal and non-systematic.

Statistical methods: SPSS/PC statistical software (version 23.0; IBM Corp., Armonk, NY, United States) for Windows was used. Count data are presented in the form of n (%), and the differences between—groups are analyzed using chi square tests, such as gender, degree and cause of anemia, etc. Visual (histogram and probability plot) and analytical (Kolmogorov Smirnov/Shapiro Wilk test) methods are used to evaluate whether variables follow a normal distribution. The measurement data that conforms to the normal distribution is represented in the form of mean ± standard deviation (SD), and independent sample *t*-test is used to determine the difference between the two groups; Paired *t*-test was used for intra group comparison before and after. For example, age, BMI, and RBC parameter scores. Non-normally distributed data are represented by median and interquartile range, and Mann—Whitney U test is used for inter group comparison; Wilcoxon signed rank test was used for intra group comparison before and after. For example, CD8^+^ T-cell levels before and after treatment. The statistical significance is set to *P* < 0.05. To further explore potential factors affecting treatment efficacy, univariate logistic regression analysis was employed to screen variables. Variables with *P* < 0.10 were included in a multivariate logistic regression model for adjustment. Regression results were expressed as odds ratios (OR) and their 95% confidence intervals (95%CI).

## Results

Of the initially screened IDA patient records during the study period, 13 patients were excluded due to incomplete key laboratory or treatment data, resulting in 80 eligible patients (40 per group) included in the final analysis.

A total of 80 IDA patients were included, including 36 males and 44 females; Age range: 22–75 years old, 48.2 ± 12.2 years old; 40 cases received treatment with Guipiheji (Guipi Mixture) combined with ferrous succinate tablets (observation group), and 40 cases received treatment with ferrous succinate tablets (control group). The proportion of patients with common comorbidities (e.g., gastrointestinal disease: 6 vs. 5) and use of concurrent medications (e.g., proton pump inhibitors: 5 vs. 4; vitamin C supplementation: 12 vs. 10) were similar between—groups. Nutritional indicators (BMI) did not differ significantly. No patient received erythropoiesis-stimulating agents or blood transfusions during the study period.

All 40 patients in the observation group were diagnosed with TCM syndromes appropriate for Guipiheji (Guipi Mixture) therapy: 25 patients (62.5%) presented with qi-blood deficiency, and 15 patients (37.5%) presented with heart-spleen deficiency. In the control group, the distribution was similar: 27 patients (67.5%) with qi-blood deficiency and 13 patients (32.5%) with heart-spleen deficiency. There was no significant difference in baseline data between the two groups of patients (*P* > 0.05) (Table 3).

**TABLE 3:** Comparison of baseline data between two groups of patients.

Baseline data	Observation group (*n* = 40)	Control group (*n* = 40)	χ ^2^*/t*	*P*
Male, n(%)	16 (40.0)	20 (50.0)	0.808	0.369
Age (year), mean ± SD	49.8 ± 11.5	46.6 ± 12.9	1.192	0.237
BMI (kg/m^2^), mean ± SD	23.5 ± 3.3	22.9 ± 3.1	0.869	0.388
Severity of anemia, n(%)			0.270	0.874
Mild	10 (25.0)	9 (22.5)		
Moderate	26 (65.0)	28 (70.0)		
Severe	4 (10.0)	3 (7.5)		
Causes of anemia, n(%)			1.588	0.662
Renal anemia	15 (37.5)	16 (40.0)		
Anemia during pregnancy	1 (2.5)	3 (7.5)		
Cancer-related anemia	4 (10.0)	5 (12.5)		
Others	20 (50.0)	16 (40.0)		
Hypertension, n (%)	9 (22.5)	8 (20.0)	0.082	0.774
Diabetes, n (%)	5 (12.5)	6 (15.0)	0.125	0.723
Gastrointestinal disorder, n (%)	6 (15.0)	5 (12.5)	0.125	0.723
Proton pump inhibitors, n (%)	5 (12.5)	4 (10.0)	0.152	0.697
Vitamin C supplementation, n (%)	12 (30.0)	10 (25.0)	0.270	0.603
Other hematinic agents, n (%)	0 (0)	0 (0)	–	–
Nutritional guidance received, n (%)	40 (100)	40 (100)	–	–
TCM syndrome, n(%)			0.240	0.624
Qi-blood deficiency	25 (62.5)	27 (67.5)		
Heart-spleen deficiency	15 (37.5)	13 (32.5)		

SD, standard deviation; BMI, Body Mass Index.

Treatment adherence: Based on pharmacy records and clinical documentation, the medication possession ratio (MPR) for ferrous succinate tablets was > 85% in both groups. In the observation group, the adherence to Guipiheji (Guipi Mixture) was similarly high (> 85%), and no major deviations from the prescribed dosage or treatment duration were documented. There was no significant difference in adherence rates between the two groups (*P* > 0.05).

**FIGURE 1:**
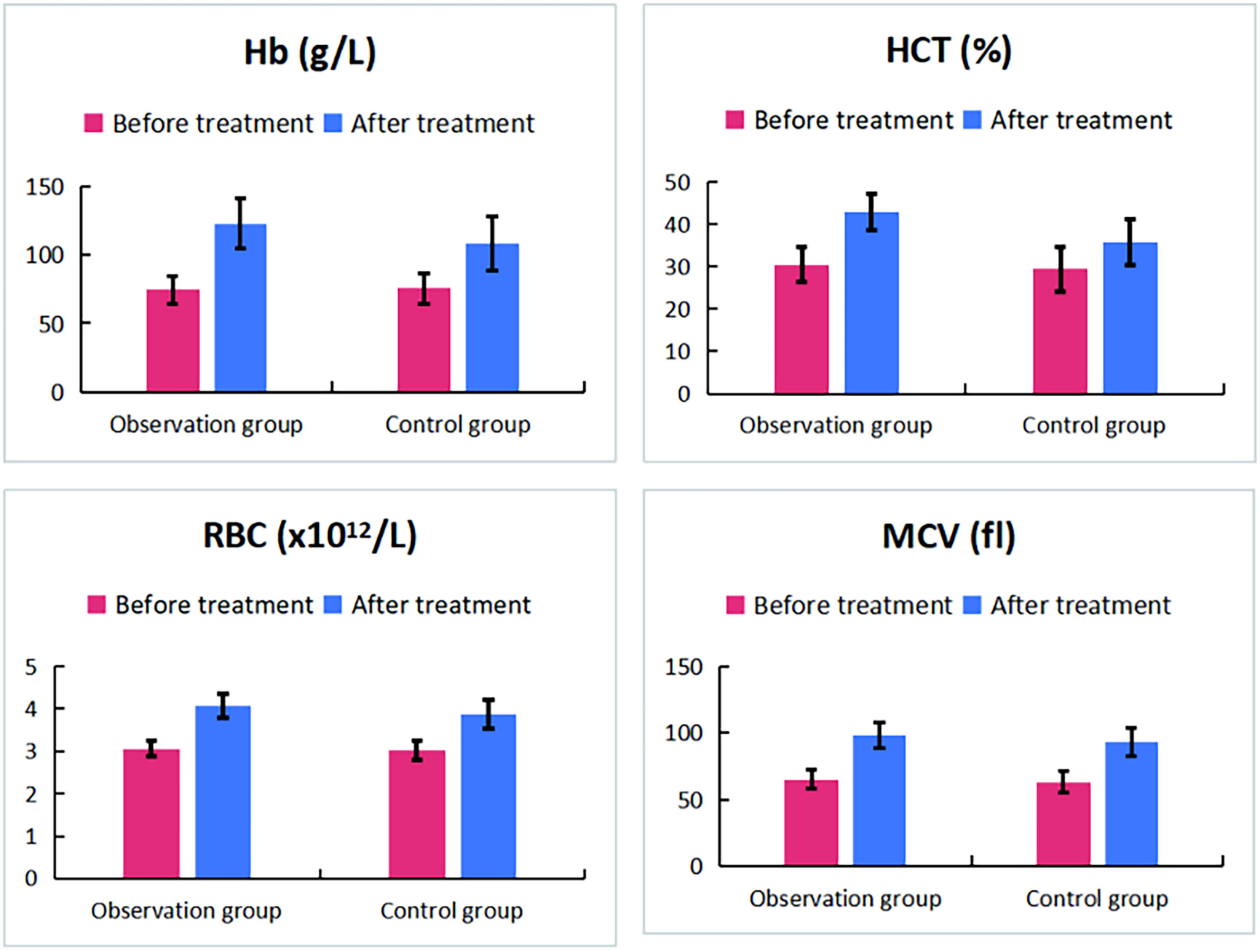
Comparison of red blood cell parameters between two groups of patients before and after treatment.

**FIGURE 2:**
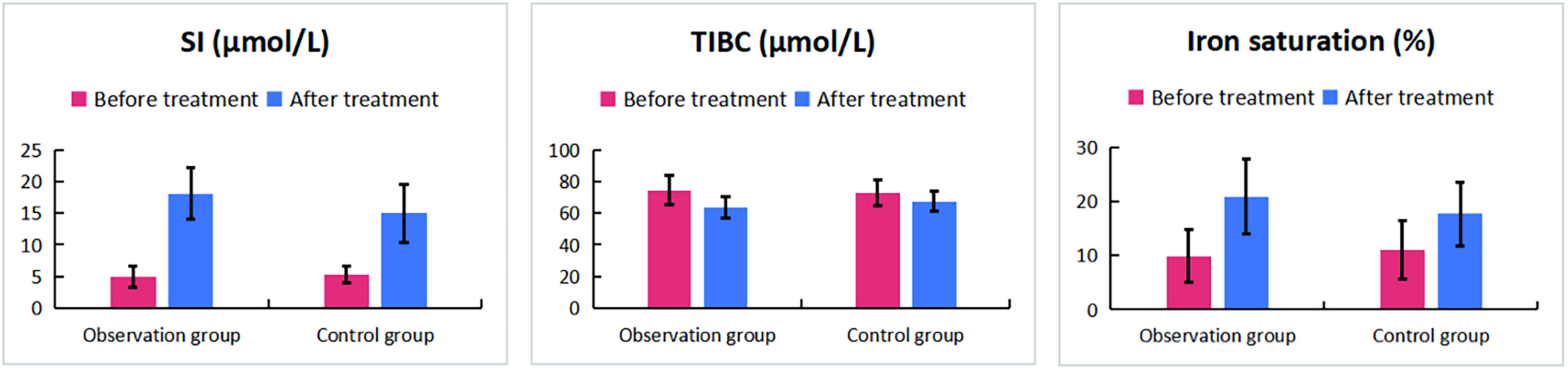
Comparison of iron metabolism indicators between two groups before and after treatment.

**FIGURE 3:**
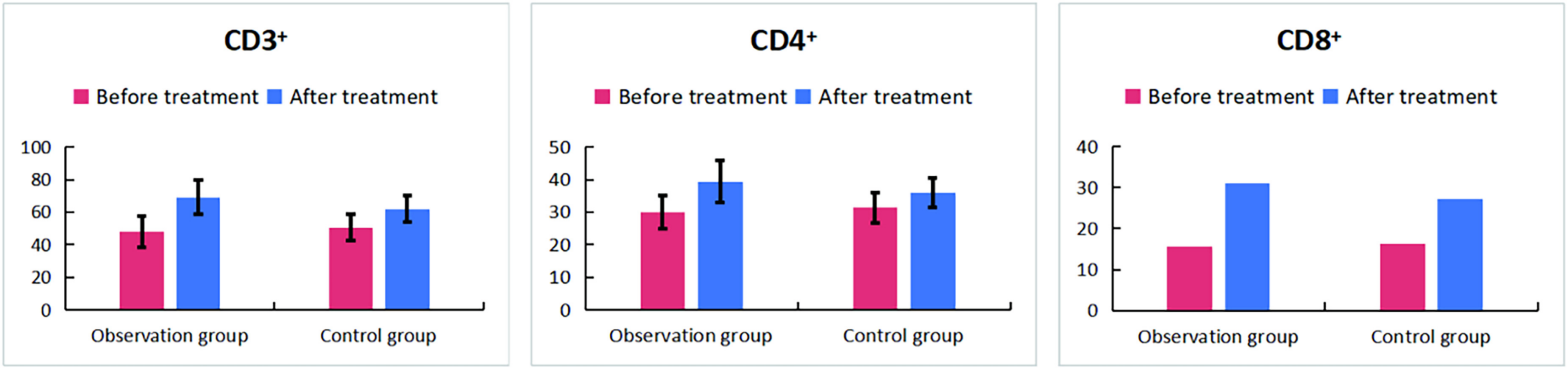
Comparison of CD3^+^, CD4^+^, and CD8^+^ T-cell levels between two groups (%).

The key hematologic, iron metabolism, and immunologic outcomes are detailed in Tables 1, 4–7. In summary, compared to ferrous succinate monotherapy, the addition of Guipiheji (Guipi Mixture) was associated with a significantly higher objective response rate (92.5% vs. 75.0%; *P* = 0.034; Table 1) and greater improvements in all measured erythrocyte parameters (Hb, HCT, RBC, MCV; all *P* < 0.05; Table 4 and Figure 1). The combination therapy also led to a more favorable iron metabolism profile, with higher serum iron and iron saturation but lower total iron-binding capacity post-treatment (Table 5 and Figure 2). Furthermore, patients receiving Guipiheji (Guipi Mixture) exhibited a greater increase in CD3^+^, CD4^+^, and CD8^+^ T-cell subsets (Table 6 and Figure 3). The incidence of adverse reactions was comparable between the two groups (22.5% vs. 15.0%, *P* = 0.390; Table 7).

**TABLE 4:** Comparison of red blood cell parameters between two groups of patients before and after treatment.

Index	Observation group (*n* = 40)	Control group (*n* = 40)	*t*	*P*
**Before treatment**				
Hb (g/L)	74.5 ± 10.1	75.6 ± 11.2	-0.440	0.661
HCT (%)	30.5 ± 4.1	29.4 ± 5.3	1.049	0.298
RBC (× 10^12^/L)	3.06 ± 0.18	3.02 ± 0.22	0.976	0.332
MCV (fl)	65.1 ± 7.2	63.0 ± 8.3	1.221	0.226
**After treatment**				
Hb (g/L)	122.8 ± 18.1*	108.0 ± 20.0*	3.497	0.001
HCT (%)	42.8 ± 4.3*	35.7 ± 5.4*	6.465	< 0.001
RBC (× 10^12^/L)	4.07 ± 0.28*	3.87 ± 0.33*	2.917	0.005
MCV (fl)	98.2 ± 9.4*	92.9 ± 10.6*	2.374	0.020

Compared to before treatment, *P < 0.05; Hb, hemoglobin; HCT, Hematocrit; RBC, red blood cell; MCV, mean corpuscular volume.

**TABLE 5:** Comparison of iron metabolism indicators between two groups before and after treatment.

Index	Observation group (*n* = 40)	Control group (*n* = 40)	*t*	*P*
**Before treatment**				
SI (μmol/L)	4.95 ± 1.6	5.28 ± 1.36	–1.009	0.316
TIBC (μmol/L)	74.5 ± 9.3	72.8 ± 8.1	0.841	0.403
Iron saturation (%)	9.95 ± 4.84	11.14 ± 5.41	–1.035	0.304
**After treatment**				
SI (μmol/L)	18.1 ± 4.1*	15.0 ± 4.6*	3.228	0.002
TIBC (μmol/L)	63.8 ± 6.6*	67.4 ± 6.5*	–2.396	0.019
Iron saturation (%)	20.9 ± 6.9*	17.7 ± 5.9*	2.257	0.027

Compared to before treatment, *P < 0.05; SI, serum iron; TIBC, total iron-binding capacity.

**TABLE 6:** Comparison of CD3^+^, CD4^+^, and CD8^+^ T-cell levels between two groups (%).

Index	Observation group (*n* = 40)	Control group (*n* = 40)	*t/Z*	*P*
**Before treatment**				
CD3^+^	47.9 ± 9.6	50.7 ± 8.3	–1.401	0.165
CD4^+^	30.0 ± 5.2	31.4 ± 4.6	–1.273	0.207
CD8^+^	15.5 (14.05–17.7)	16.2 (14.5–19.7)	–	0.233
**After treatment**				
CD3^+^	69.2 ± 10.3*	62.0 ± 7.9*	3.501	0.001
CD4^+^	39.3 ± 6.4*	36.0 ± 4.6*	2.588	0.012
CD8^+^	30.95 (26.4–33)*	27.25 (19.5–32.05)*	–	0.012

Compared to before treatment, *P < 0.05; –: Mann—Whitney U test.

**TABLE 7:** Comparison of adverse reaction rates between two groups.

Index	Observation group (*n* = 40)	Control group (*n* = 40)	*P*
Nausea	3 (7.5)	1 (2.5)	–
Epigastric discomfort	2 (5.0)	2 (5.0)	–
Phlebitis	1 (2.5)	1 (2.5)	–
Loss of appetite	2 (5.0)	1 (2.5)	–
Facial flushing	1 (2.5)	0 (0)	–
Constipation	0 (0)	1 (2.5)	–
Total number of occurrences	9 (22.5)	6 (15.0)	0.390

### Analysis of influencing factors

The results of the univariate logistic regression analysis showed that the severity of anemia (OR = 3.595, 95%CI: 1.139–11.350, *P* = 0.029) and pre-treatment hemoglobin level (OR = 0.933, 95%CI: 0.874–0.996, *P* = 0.037) were significantly associated with ORR. Other variables such as gender, age, BMI, and comorbidities showed no statistical significance (*P* > 0.05). After adjusting for the aforementioned variables with *P* < 0.10 (anemia severity, pre-treatment Hb, pre-treatment MCV) in the multivariate logistic regression model, none of the variables demonstrated independent predictive value (*P* > 0.05), as detailed in Table 8.

**TABLE 8:** Results of Logistic Regression Analysis for Factors Affecting ORR.

Variable	Univariate analysis	*P*	Multivariate analysis	*P*
	OR (95%CI)		OR (95%CI)	
Anemia severity	3.595 (1.139–11.350)	0.029	1.609 (0.250–10.363)	0.617
Pre-treatment Hb	0.933 (0.874–0.996)	0.037	0.954 (0.861–1.057)	0.368
Pre-treatment MCV	0.932 (0.856–1.015)	0.107	0.944 (0.865–1.031)	0.201
Constant	–	–	3722.147	0.212

## Discussion

This retrospective study demonstrates that adding Guipiheji (Guipi Mixture) to conventional ferrous succinate therapy led to several clinically meaningful improvements over iron monotherapy. Most notably, the combination therapy resulted in a substantially higher objective response rate (92.5% vs. 75.0%) and greater increases in hemoglobin (mean increase ≈ 48.3 g/L vs. ≈ 32.4 g/L) and serum iron. These differences suggest that the integrated approach not only benefits more patients but also achieves a faster or more complete correction of anemia—an important practical consideration given the debilitating symptoms and functional impairment associated with IDA. Furthermore, the combination was associated with a more favorable iron metabolism profile and improved T-cell immunity, without increasing adverse events. Collectively, these preliminary clinical findings are consistent with the notion that Guipiheji (Guipi Mixture), in combination with iron supplementation, may address multiple clinical facets of IDA more effectively than iron alone. This potential synergy warrants further investigation but should not be construed as a proven mechanistic interaction based on the present study design.

The results of this study show that compared with pure ferrous succinate tablets, combination therapy with Guipiheji (Guipi Mixture) and ferrous succinate tablets yields superior efficacy in improving IDA, validating our initial hypothesis. This validates our initial hypothesis and aligns with the findings of the research results of Huang et al. (9) and Wu et al. (10), both of which confirm the therapeutic effect of Guipiheji (Guipi Mixture) on IDA. As is well known, oral iron supplements are one of the most common clinical practices for treating IDA (5, 11). It has the advantages of low price, simple administration, and high safety (11). Unfortunately, the interaction between ferritin and iron transporters can easily lead to inflammation and inhibition of absorption; Moreover, there are defects such as gastrointestinal adverse reactions, absorption disorders, slow correction of anemia, and poor compliance (6, 12). TCM believes that the spleen and stomach are the source of qi and blood biochemistry. Anemic patients often suffer from nutrient absorption disorders due to weak spleen and stomach (even if iron supplements are supplemented, the therapeutic effect is poor due to poor absorption) (13). TCM formulas optimize the “basic soil” for nutrient absorption by strengthening the spleen and nourishing qi components. Lv et al. (14) conducted a meta-analysis of 13 studies and found that Guipi decoction can quickly alleviate the symptoms of anemia patients, demonstrating definite therapeutic effects and improving their quality of life.

The observed clinical benefits may be contextualized within the reported pharmacological activities of the formula’s constituents, though the present study was not designed to validate specific mechanistic pathways. The main herbal components of Guipiheji (Guipi Mixture), such as Panax ginseng f. angustifolius (Burkill) H.L.Li [Asteraceae], Atractylodes macrocephala Koidz. [Asteraceae], and Astragalus mongholicus Bunge [Fabaceae], have been independently studied for their hematinic and immunomodulatory properties in prior literature. For example, previous research suggests that ginsenosides from Panax ginseng f. angustifolius (Burkill) H.L.Li [Asteraceae]) may stimulate hematopoietic organs and promote bone marrow stem cell proliferation (14, 15). Similarly, evidence from other studies indicates that polysaccharides and flavonoids from Astragalus mongholicus Bunge [Fabaceae] could enhance the hematopoietic microenvironment and modulate immune function (16), while components of Atractylodes macrocephala are reported to support gastrointestinal function and nutrient absorption (17). Therefore, it is plausible to speculate that the combined action of these constituents may contribute to the superior outcomes seen in the observation group, although direct mechanistic confirmation requires future targeted investigation.

In our study, after treatment, the levels of Hb, HCT, RBC, and MCV in the observation group were significantly higher than those in the control group. This indicates that oral administration of ferrous succinate tablets can significantly improve RBC levels. This is consistent with the research results of Huang et al. (9). Although oral iron supplements are convenient to take, they have absorption barriers and slow correction of anemia. Mechanistically, previous experimental studies have proposed that components within Guipiheji (Guipi Mixture), such as Angelica sinensis, may promote bone marrow microcirculation and enhance nutrient supply to hematopoietic cells (14, 18). Additionally, polysaccharides from Atractylodes macrocephala have been reported to improve red blood cell membrane stability (17, 19). Drawing from these prior findings, one may hypothesize that Guipiheji (Guipi Mixture), when combined with ferrous succinate, could potentially improve the hematopoietic microenvironment and intestinal iron absorption. For instance, experimental studies have suggested that Astragalus membranaceus polysaccharide may enhance intestinal iron transporter activity (20), which could theoretically facilitate better iron uptake in IDA patients with compromised absorption. While our clinical outcomes are consistent with such a possibility, it is important to emphasize that the present study was not designed to validate these specific molecular pathways, and the proposed mechanisms remain speculative. In addition, IDA patients experience immune microenvironment imbalance (such as T-cell dysfunction) due to iron deficiency and insufficient qi and blood. After treatment, the improvement of the immune microenvironment in the observation group was better than that in the control group. From a traditional Chinese medicine perspective, Guipiheji (Guipi Mixture) is a classic formula for nourishing qi and blood, and its herbal components (e.g., Codonopsis pilosula (Franch.) Nannf. [Campanulaceae], Angelica sinensis (Oliv.) Diels [Apiaceae], Astragalus mongholicus Bunge [Fabaceae], Atractylodes macrocephala Koidz. [Asteraceae]) have been reported in prior literature to possess immunomodulatory properties. Thus, it is conceivable that the formula might contribute to immunologic improvement observed in the combination group, though this study does not provide direct evidence for a specific immunomodulatory mechanism of Guipiheji (Guipi Mixture).

The inclusion of T-cell subset analysis was based on established evidence that iron deficiency directly impairs T-cell proliferation and function, contributing to immune dysregulation in IDA (21). Therefore, we included these parameters as exploratory biomarkers of systemic immune-metabolic status. After treatment, increases in CD3^+^, CD4^+^, and CD8^+^ T-cell percentages were observed in both groups, with a greater numerical increase in the combination group (Table 6). These shifts are consistent with the expected immune recovery following iron repletion. The more pronounced immunologic changes in the Guipiheji (Guipi Mixture)-added group may be interpreted within the framework of TCM theory as reflecting a broader systemic improvement consistent with the formula’s traditional “qi-tonifying and blood-nourishing” properties. However, it must be clearly stated that this study provides no direct evidence for a specific immunomodulatory mechanism of Guipiheji (Guipi Mixture), and the observed immune parameter changes could also be secondary to more efficient iron repletion or other unmeasured factors. Therefore, these immunologic findings should be interpreted as preliminary and descriptive, complementing the primary hematologic outcomes.

In terms of safety, there was no significant difference in the overall incidence of adverse reactions between the two groups. This indicates that adding Guipiheji (Guipi Mixture) on the basis of oral ferrous succinate tablets is safe. The main components of Guipiheji [e.g., Codonopsis pilosula (Franch.) Nannf. [Campanulaceae], Angelica sinensis (Oliv.) Diels [Apiaceae], Astragalus mongholicus Bunge [Fabaceae], Atractylodes macrocephala Koidz. [Asteraceae]) are all low toxicity traditional Chinese medicinal materials recorded in the Chinese Pharmacopoeia, and there are no clear liver and kidney toxicity components (such as alkaloids and heavy metals). Similarly, Jeong et al. (22) conducted a meta-analysis of 28 studies and found that adding herbal medicines such as Angelica sinensis (Oliv.) Diels [Apiaceae], Astragalus mongholicus Bunge [Fabaceae] and Atractylodes macrocephala Koidz. [Asteraceae] to oral iron supplements alone is safe and is associated with fewer adverse reactions. However, in our study, the observation group did not show a higher advantage in reducing adverse reactions. It should be acknowledged, however, that the statistical comparison of adverse reactions may be underpowered due to the relatively small number of events in this cohort. The non-significant *P*-value does not necessarily imply equivalence in safety profiles, and larger prospective studies are needed to more reliably assess potential differences in adverse event rates between the two regimens.

This study employed logistic regression to analyze potential factors influencing patients’ achievement of the objective response rate (ORR). Univariate analysis indicated that a more severe baseline anemia (OR = 3.595, *P* = 0.029) and a lower pre-treatment hemoglobin level (OR = 0.933, *P* = 0.037) were significant predictive factors for patients attaining treatment response. This finding has a clear clinical logic: patients with more severe baseline anemia have a greater relative potential for their hemoglobin levels to meet the “response” criteria (e.g., an Hb increase > 15 g/L or > 30 g/L) after treatment, thus being statistically more likely to be classified as treatment responders (ORR). However, after adjustment in the multivariate analysis, the independent predictive effects of these factors did not reach statistical significance (*P* > 0.05). This may be partly due to the limited sample size, resulting in insufficient statistical power for the multivariate model. On the other hand, it also suggests that anemia severity and pre-treatment Hb may not be completely independent predictors; their effects might be confounded or mediated by each other or by other clinical features not fully measured (such as the completeness of iron store depletion or coexisting inflammatory status).

More importantly, this analytical result enhances the credibility of this study’s main conclusion from another perspective. Since the univariate analysis suggested that “more severe anemia” and “lower Hb” were predictors of treatment response, then if the higher ORR in the observation group (the combination therapy group) were merely due to its patients having more severe baseline conditions, we should have observed systematic differences between the two groups in these factors in the baseline data. However, as shown in Table 3, there were no statistically significant differences between the two groups in key baseline indicators such as pre-treatment Hb and anemia severity grade (*P* > 0.05). Therefore, the significantly higher ORR in the observation group (92.5% vs. 75.0%) is unlikely to be explained by differences in patients’ baseline conditions, but is more likely attributable to the synergistic therapeutic effect of Guipiheji (Guipi Mixture) combined with ferrous succinate tablets itself. Future prospective studies should include more comprehensive iron metabolism and inflammatory indicators in larger samples to construct a more robust predictive model for IDA treatment response.

### Limitations of the study

Several limitations of this study should be acknowledged: (1) As a single-center retrospective analysis, the interpretability of our findings is constrained by inherent limitations of the observational design. The non-random allocation of adjunctive Guipiheji (Guipi Mixture) may have been subject to selection and indication bias. For instance, clinicians might have been more inclined to add Guipiheji (Guipi Mixture) for patients with more pronounced TCM syndromes (e.g., severe fatigue or poor appetite) or those perceived as having poorer tolerance to iron monotherapy. Although we attempted to balance measured baseline characteristics, unmeasured or inadequately documented confounders (such as subtle differences in symptom severity, patient preferences, or clinician prescribing habits) could residually influence the outcomes. (2) We did not control or systematically record participants’ detailed dietary intake during the 8-week intervention. Variations in the consumption of iron-rich foods (e.g., red meat, spinach), enhancers (e.g., vitamin C-rich fruits), or inhibitors (e.g., tea, coffee, calcium supplements) of iron absorption represent a significant unmeasured confounding factor that could differentially affect hematological and iron metabolism outcomes in both groups. (3) The absence of longer-term follow-up data limits our understanding of the durability of the treatment effect, the optimal treatment duration, and the long-term recurrence rate of IDA after discontinuing therapy. This precludes any assessment of whether the combination therapy merely accelerates correction or also sustains remission more effectively. (4) Inflammatory markers (e.g., hs-CRP, IL-6) were not collected in this study. Given the documented interplay between iron deficiency, chronic inflammation, and immune dysfunction, the absence of this data limits our ability to mechanistically interpret the observed improvements in T-cell subsets and to disentangle whether the immunomodulatory effect is secondary to improved iron status or a direct anti-inflammatory action of the herbal formula. (5) Important confounders (severity of anemia, comorbidities, inflammation, taking other medications, menstrual blood loss, GI absorption issues, renal function, socioeconomic variables, etc.) were not controlled. (6) Dietary intake, which can influence iron absorption and hematological outcomes, was not controlled. (7) While we have provided the standardized composition of Guipiheji (Guipi Mixture), we were unable to present batch-specific phytochemical fingerprinting data for the commercial preparation used. Although quality is regulated by GMP, inherent variability in the content of active compounds (e.g., polysaccharides, saponins) across batches could influence therapeutic potency and affect the reproducibility of the study findings from a pharmacognostic perspective.

## Conclusion

This retrospective study provides preliminary evidence that adding Guipiheji (Guipi Mixture) to ferrous succinate tablets may improve hematologic and iron metabolism outcomes in IDA patients, without an apparent increase in short-term adverse reactions. Given the inherent limitations of observational designs, such as potential unmeasured confounding and selection bias, these findings should be interpreted as hypothesis-generating. To confirm these results and establish causal efficacy, prospective, randomized controlled trials with larger sample sizes, longer follow-up, and rigorous control of confounding factors (e.g., dietary intake) are strongly recommended.

## Data Availability

The original contributions presented in this study are included in this article/supplementary material, further inquiries can be directed to the corresponding author.
